# Interprofessional Education: A Systematic Review of Educational Methods in Postgraduate Health Professions Programs

**DOI:** 10.1111/tct.70114

**Published:** 2025-06-19

**Authors:** Nouf Sulaiman Alharbi, Lujayne Bukhari, Noof Khalid Albaz, Abdulrahman Saleh Alraddadi, Reema Albilehi, Reem Alkahtani, Seema Nasser, Taghreed Alnahedh, Marwh Gassim Aldriwesh

**Affiliations:** ^1^ Department of Medical Education College of Medicine, King Saud bin Abdulaziz University for Health Sciences Riyadh Saudi Arabia; ^2^ King Abdullah International Medical Research Center Riyadh Saudi Arabia; ^3^ Ministry of the National Guard ‐ Health Affairs Riyadh Saudi Arabia; ^4^ Department of Pediatrics Prince Sultan Military Medical City Riyadh Saudi Arabia; ^5^ Department of Basic Medical Sciences College of Medicine, King Saud bin Abdulaziz University for Health Sciences Riyadh Saudi Arabia; ^6^ English Department College of Science and Health Professions, King Saud bin Abdulaziz University for Health Sciences Riyadh Saudi Arabia; ^7^ Department of Nursing College of Nursing, King Saud bin Abdulaziz University for Health Sciences Riyadh Saudi Arabia; ^8^ Department of Clinical Laboratory Sciences College of Applied Medical Sciences, King Saud bin Abdulaziz University for Health Sciences Riyadh Saudi Arabia

**Keywords:** educational approaches, interprofessional education, learning approaches, multiprofessional education, postgraduate medical education, teaching approaches

## Abstract

**Background:**

Several studies on interprofessional education (IPE) explored student's knowledge acquisition, teamwork skills and collaborative behaviours. However, the approaches to teaching and learning IPE remain underresearched and reported especially at the postgraduate level. This systematic review aimed to establish how IPE has been implemented at the postgraduate level among different health professions, focusing on teaching and learning approaches.

**Methods:**

The systematic review was conducted in 2022–2025. It utilized three carefully identified databases: PubMed, ScienceDirect and the Cochrane Library. Publications were included after being screened based on a clear protocol and preidentified eligibility criteria. The research team used CADIMA software to screen articles published from 2010 to 2025.

**Results:**

Thirty‐seven articles were considered in this systematic review. These articles were mainly from the United States, United Kingdom and Canada. Various educational approaches and a wide variety of tools were utilized to deliver IPE among health professionals at the postgraduate level. Yet, the findings indicated that simulation was at the top of the used approaches. This systematic review also revealed that IPE activities at the postgraduate level need to be focused more on interprofessional role learning and dual identity development.

**Conclusions:**

The evidence synthesized in the current systematic review reveals that educators prefer simulation to deliver IPE activities. Yet, the evidence calls for more focused planning for IPE activities at the postgraduate level to advance IPE delivery and ensure targeting immersion and mastery levels of IPE development.

**Abbreviations**
IPE: interprofessional education
UBC: University of British Columbia

## 1 Background

Interprofessional education (IPE) is a pedagogical approach that enables learners to work collaboratively [1]. In healthcare, IPE occurs when two or more learners from different health professions learn about, from and with each other to promote collaboration and improve healthcare outcomes [1]. IPE has been implemented widely in many countries in both undergraduate and postgraduate curricula. The term undergraduate indicates that the IPE activities are designed to enhance competencies among college or university students who are enrolled in initial degree, typically leading to a bachelor's degree in health sciences. Conversely, the term postgraduate indicate that the activities are designed at a more advanced level that suits learners who already completed their bachelor's degrees and are mostly practicing in a clinical setting, this group may also include students enrolled in academic programmes such as masters or high diploma or professional training such as continuous professional development activities [2]. Consequently, IPE outcomes can vary significantly between undergraduate and postgraduate students due to differences in experience, readiness and educational contexts [2, 3]. Regardless of the participants level, most health professions employed in IPE are nursing and medicine [2]. Moreover, a previous study by Greer and colleagues that examined the prevalence and nature of IPE in several academic centres in the United States indicated that IPE experiences are mainly offered to undergraduate students [4]. On the contrary, an international scan was commissioned by the World Health Organization to inform efforts to support IPE globally revealed that only 16% of published interprofessional curricula were for postqualification years [5]. Since then, the need for better implementation of IPE in postgraduate training was recognized, and it has been recommended by the Accreditation Council for Graduate Medical Education and the American Society of Health‐System Pharmacists to integrate interprofessional patient care during residency training [6].

According to the Interprofessional Education Collaborative (IPEC) group, which represents 21 health professions associations committed to ensuring that health professionals are skilled in patient‐centred, community‐oriented, interprofessional, collaborative practice [7], educators need to consider four main competencies when planning and designing IPE curricula. These competencies are as follows: (1) supporting and providing mutual respect and shared values; (2) functioning effectively as an interprofessional group/team; (3) recognizing and realizing roles and responsibilities of each team member; and (4) communicate effectively to achieve the team approach. Moreover, educators should consider accommodating the varying roles of participants, their competencies and collaborative skills needed across disciplines, to ensure effective teamwork and patient‐centred care, which would require the utilization of various interactive teaching and learning approaches [8].

Additionally, two models are important and can aid educators to better understand both the cognitive and social dimensions of IPE, the model developed by the University of British Colombia (UBC) and the IPE socialization framework [9, 10]. Firstly, the UBC model provides an understanding of the learning processes involved in IPE. It consists of three phases, that is, exposure, immersion and mastery. In the exposure phase, learners are expected to develop an understanding of their own profession while also becoming aware of the roles played by other professions. During the immersion phase, learners are required to acquire essential knowledge and skills pertinent to their specific fields. They are also introduced to other professions through their clinical placements. Finally, in the mastery phase, practitioners who have ‘mastered’ interprofessional concepts are anticipated to integrate them into their daily practice. At this stage, practitioners should possess a comprehensive understanding of both their own profession and those of others [9].

Secondly, the IPE Socialization Framework was proposed by Khalili and his colleagues to highlight the significance of interprofessional socialization, particularly during the early stages of training. Just like the UBC model, the process outlined three distinct stages [10]. First, learners confront their uniprofessional identities by engaging in situations that challenge misconceptions about their roles. They must also address negative perceptions held by professionals from other fields. These challenges encourage learners to seek solutions and adopt an open‐minded approach towards other professions by learning about, with and from their peers. This stage is crucial for dismantling barriers that can impede cooperation and collaboration. As learners progress to the second stage, the IPE environment begins to thrive. During this stage, learners start to develop IPE competencies, such as understanding the roles of other professions, by collaborating in interprofessional teams to work on simulated patients. Finally, the last stage marks the full development of a dual professional identity. At this point, learners enhance their understanding of their own professional roles while also fostering a sense of belonging within the interprofessional community [10].

A recent systematic review reported the teaching and learning approaches utilized to deliver the IPE at the undergraduate level in 16 published studies. The review showed that simulation‐based education, e‐learning and problem‐based learning were the most prevalent approaches used to deliver IPE in undergraduate health professions education [11]. In addition, the review has utilized both the UBC model and the IPE socialization framework to reflect on the results and indicated that the majority of approaches utilized in undergraduate IPE targeted the exposure phase and uniprofessional identity. However, there is a lack of similar reflection focusing on teaching and learning strategies implemented in IPE at the postgraduate level. Yet, previous studies have shown that IPE implementation is more comprehensive than clinical courses in postgraduate health professions. In other words, IPE can be applied in research, leadership, management, quality improvement, deliberation and bioethics courses. Nevertheless, IPE's teaching and learning method must be compatible with the course's nature and content.

The literature needs to indicate more comprehensive evidence of teaching and learning approaches used for IPE at postgraduate level. Such evidence is important to support educators while designing IPE activities that target sustaining improvements in attitudes towards teamwork and collaboration over longer period and integrating IPE skills into practice. Moreover, such evidence is essential to address the variability in how IPE is implemented across different settings and disciplines. Also, it can help identify best practices and align teaching methods with key competencies, including teamwork, communication and role clarity, which are crucial for effective interprofessional collaboration (IPC) [12]. Considering that, we aimed to systematically review the literature and identify teaching and learning approaches used to deliver IPE for postgraduate learners. The following systematic review provides essential context for interpreting the outcomes of IPE teaching and learning approaches.

## 2 Methods

This systematic review was developed using AMEE guide 94, titled (a practical approach to systematic reviews in medical education) [13]. The research team comprised 11 dedicated academics from various health professions who brought unique skills and experiences to the study. Their collective knowledge covered the conduction of systematic reviews, IPE and several areas in health professions education such as curriculum development, utilization of different educational strategies, assessment and quality. The involvement of these individuals ensured that the review was conceived from multiple perspectives, making it more comprehensive and well rounded. Moreover, the team consulted with a librarian during the process of selecting which databases to include and what terms or strings would be most appropriate to yield articles relevant to the study and answer the research question.

To ensure systematic progress, the team created a detailed protocol to guide the process of scanning and reviewing documents and to track their progress to ensure the quality of the work being performed. The team utilized the Participants, Educational Aspects and Outcomes (PEO) model to formulate a specific question for the current systematic review. Explicitly, the team clarified the following details: who the participants were (postgraduate students in healthcare professions), what the educational aspect was (IPE) and what the outcome was (teaching and learning approaches for IPE). It is worth noting here that educational approaches and tools are interconnected; tools serve as instruments within an approach to help learners achieve desired outcomes. In other words, teaching and learning approaches use tools to achieve desired outcomes [14].

The research team consulted with a librarian to select the most appropriate databases and search strings. Three databases, including PubMed, Cochrane Library and ScienceDirect, were selected and searched from January 2010 to January 2025 using eight search strings as follows:

**Table:** 

Interprofessional education	OR	multi‐professional education	AND	postgraduate
Interprofessional education	OR	multi‐professional education	AND	post‐licensure
Interprofessional education	OR	multi‐professional education	AND	post ‐specialization.
Interprofessional education	OR	multi‐professional education	AND	residency
Interprofessional learning	OR	Interprofessional learning	AND	postgraduate
Interprofessional learning	OR	Interprofessional learning	AND	post‐licensure
Interprofessional learning	OR	Interprofessional learning	AND	post ‐specialization.
Interprofessional learning	OR	Interprofessional learning	AND	residency

Supporting Information S1 shows the search strategy for identification of articles for the current systematic review.

As part of the research protocol, the inclusion criteria for the systematic review were clearly defined. Eligible studies had to be published in English and focus on IPE activities designed for postgraduate learners from health professions including medicine, dentistry, nursing, pharmacy and allied health sciences, which included nutrition, occupational therapy, physical therapy, audiology, speech pathology, respiratory therapy and radiology. The studies needed to explicitly report the use of at least one teaching method related to IPE activities. Only studies examining educational activities were included, while those focusing on communication, collaborative work or teamwork were excluded. Furthermore, only peer‐reviewed journal articles were considered for inclusion, and secondary data, reviews and reflective articles were not taken into account. A comprehensive screening process was carried out to ensure the methodological integrity and relevance of the studies included in the review.

To enhance the precision of the current study and mitigate the impact of random error and bias, a collaborative approach was adopted and CADIMA (https://www.cadima.info/index.php), which is a free web tool, was utilized to facilitate the screening processes for articles and documentation for decisions of the research team members. From the members (A.S.R., L.B., R.A., R.A., S.N., T.A., N.A. and N.S.A.), at least two different team members screened all the titles and abstracts, and consensus was required to exclude an article. Instances lacking sufficient details prompted the advancement to the following screening stage, where the full text was reviewed by both members for a conclusive decision on inclusion or exclusion. Any discrepancies between the two reviewers were resolved through consultation with a third team member to attain consensus. Thorough documentation of exclusion justifications was maintained throughout the article selection stages. The PRISMA flow diagram was used to visually represent the systematic search and selection processes for primary articles in the review. At this point, articles meeting the inclusion criteria were included irrespective of their quality, while the assessment of article's quality occurred in the subsequent step.

Two independent team members conducted the extraction of data and quality assessments (L.B. and N.S.A.), with any discrepancies resolved by a third reviewer to ensure consensus. Given the specific focus of this systematic review, descriptive data were obtained using a purpose‐built data extraction tool designed to align with the review's objectives (Supporting Information S2). This tool encompassed crucial information from each article, including the title, author(s) and publication year. Moreover, it captured details about health professionals participating in postgraduate IPE activities, along with the settings, contexts and teaching and learning approaches for IPE. In addition, the tool included information about the type of IPE activity, how it was evaluated and what findings were reported.

To check the quality of the articles included, a quality checklist adopted from Mays et al.'s checklist for mixed‐methodology case studies and other in‐depth complex designs were utilized. This checklist was originally developed in 2001 [15] (see Supporting Information S3 and S4) and incorporated criteria related to the transparency of ethical considerations, the intended objectives of IPE programmes, the processes involved in IPE implementation, the healthcare professions participating, contextual factors and funding sources. It also included assessment criteria for evaluating the IPE programmes, encompassing the clarity of study design, sampling methods, data collection, data analysis, results and conclusions.

## 3 Results

### 3.1 Search Results

**FIGURE 1:**
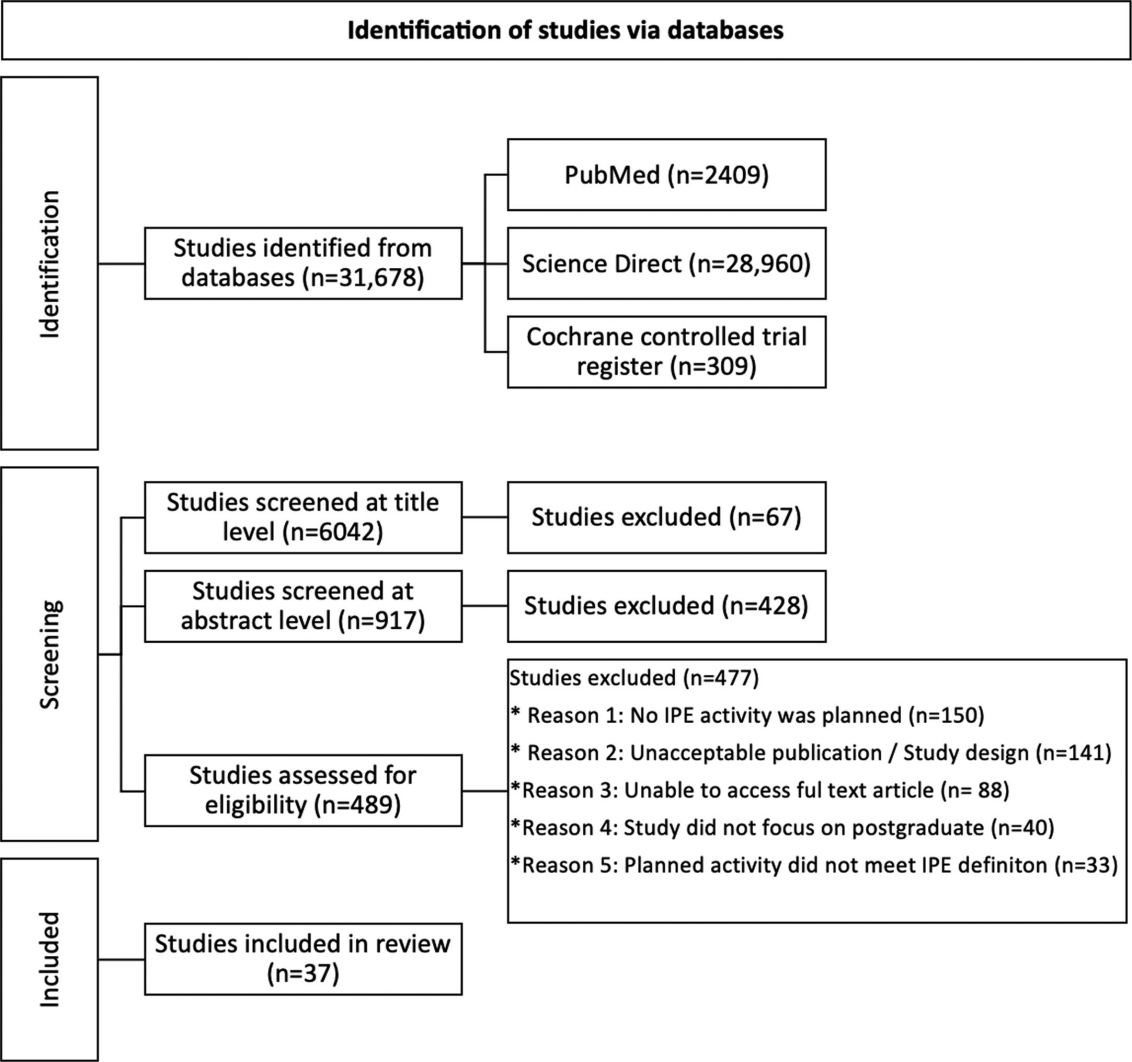
PRISMA flow chart describes the process to extract articles for inclusion in the systematic review.

A total of 31,678 articles resulted from literature search utilizing the following databases: PubMed (*n* = 2409), ScienceDirect (*n* = 28,960) and Cochrane controlled trial register (*n* = 309); see Figure 1. Of these, 25,636 were identified as duplicates, which resulted in 6959 articles that were screened at title and abstract level. Consequently, 489 articles were assessed for eligibility to be included in the present review. As a result, 477 were excluded due to reasons related to IPE activity, study design, IPE not performed at postgraduate level, or restricted access to full‐text article. Hence, a total of 37 articles met the inclusion criteria and were included in the present review [12, 16, 17, 18, 19, 20, 21, 22, 23, 24, 25, 26, 27, 28, 29, 30, 31, 32, 33, 34, 35, 36, 37, 38, 39, 40, 41, 42, 43, 44, 45, 46, 47, 48, 49, 50, 51].

### 3.2 Quality Assessment

The quality of the studies included in this review was evaluated using 15 criteria, as detailed in Supporting Information S6. Each criterion was rated as ‘Yes’, ‘No’, ‘Not Applicable’ or ‘Unclear’. The scores for the articles ranged from 5 to 14 points. Most of the studies were deemed to be of acceptable to good quality, with an average score of 10.5. Most of the included articles addressed a specific question or aim and described the context of the study and the participants. However, there was no clarification if the study was supported financially in 16 studies (43%) [16, 17, 19, 21, 24, 25, 31, 32, 33, 37, 38, 39, 44, 48, 49, 51]. In 32 studies (86%), the authors' positions and roles were unclearly explained, and the resulting bias was not considered in this review [12, 17, 18, 19, 20, 22, 23, 24, 25, 26, 27, 28, 29, 30, 31, 32, 33, 35, 37, 38, 39, 40, 41, 42, 43, 45, 46, 47, 48, 49, 50, 51]. Further, the declaration of interest was not stated in 10 studies (27%) [19, 21, 25, 26, 31, 32, 37, 38, 39, 42].

### 3.3 Findings of Included Studies

Characteristics of the included studies (*N* = 37) were summarized in Supporting Information S5 [12, 16, 17, 18, 19, 20, 21, 22, 23, 24, 25, 26, 27, 28, 29, 30, 31, 32, 33, 34, 35, 36, 37, 38, 39, 40, 41, 42, 43, 44, 45, 46, 47, 48, 49, 50, 51]. The data extracted from the literature indicated that the majority of IPE programmes were implemented in the United States (51%; *n* = 19) [17, 18, 23, 27, 30, 32, 35, 36, 37, 38, 39, 40, 41, 42, 43, 45, 46, 50, 51], followed by the United Kingdom (10.8%; *n* = 4) [16, 29, 31, 34], Canada (8%; *n* = 3) [25, 33, 47] and Taiwan (5%; *n* = 2) [20, 44]. Only one IPE programme was performed in postgraduate education in the following countries: Switzerland (2.7%; *n* = 1) [26], Romania (2.7%; *n* = 1) [21], Tanzania (2.7%; *n* = 1) [24], Brazil (2.7%; *n* = 1) [48], Denmark (2.7%; *n* = 1) [12], Netherlands (2.7%; *n* = 1) [49] and Iran (2.7%; *n* = 1) [28]. Two included studies did not indicate where the IPE postgraduate programme was conducted (5%; *n* = 2) [19, 22].

This systematic review identified a total of five learning approaches, each employing multiple tools to achieve the desired learning outcomes in postgraduate IPE programmes (see Table 1 for detailed descriptions of the approaches and the corresponding tools used under each). Notably, simulation‐based learning emerged as the most frequent learning approach reported in 23 studies (62%) [16, 17, 18, 19, 20, 21, 22, 23, 24, 25, 26, 27, 28, 29, 31, 32, 33, 34, 36, 39, 40, 41, 44]. The second most frequent approach was active learning, which appeared through the utilization of a wide variety of interactive sessions, from simple interactive lectures to more complex case‐based discussions, problem‐based or project‐based activities, accounting for 32% (*n* = 12) of the studies [12, 18, 23, 25, 28, 30, 41, 42, 43, 45, 46, 47]. Didactic learning was employed in 24% (*n* = 9) of the studies [17, 21, 30, 36, 41, 43, 46, 48, 51]. However, it is important to note that all studies that used a didactic session combined it with at least one other approach. Workshops were also used in delivering IPE for postgraduate health professionals, such as clinician–patient communication workshops, modified buzz groups and virtual workshops, which account for 14% (*n* = 5) [17, 23, 35, 42, 49]. Finally, work‐based learning appeared to be the least utilized approach (11%; *n* = 4) [46, 47, 48, 50]. It is worth noting that 14 studies (38%) had utilized a at least two approaches to deliver IPE [17, 18, 21, 23, 25, 28, 30, 36, 41, 42, 43, 46, 47, 48].

**TABLE 1:** Teaching and learning approaches implemented in the interprofessional education of the postgraduate curricula.

Learning approaches	Definition of the learning approach	IPE on studies, *n* (%)	Tools used
Simulation‐based learning	Any educational activity that employs simulation tools to imitate or recreate a clinical scenario [52]	23 (62)	Utilizing simulators Team‐based simulation (focus on team‐based approaches) High‐fidelity simulation Scenario‐based simulation Mixed simulation modality Computer‐based simulation training Role play
Active learning	Teacher‐centred approach where participants will experience a good level of interaction [53]	12 (32)	Interactive lectures IPE small group discussions Snowball interprofessional discussions Case‐based discussion Problem‐based learning In‐service practical activities Reflective practices Mentoring sessions Expert discussion Team building exercises Experiential and cooperative learning workshops Project based activities Self‐study sessions
Didactic learning	Teacher‐centred approach where students' participation is at minimum level [53]	9 (24)	Virtual lectures In‐person lectures Seminars Theoretical classes PowerPoint presentations
Workshops	Any educational activity aimed at a group of people, providing practical content tailored to their needs while promoting collaboration among participants [54]	5 (14)	Clinician–patient communication workshops Modified buzz group Virtual workshops
Work‐based learning	An educational approach that enables students to gain hands‐on experience in real‐world settings [55]	4 (11)	Clinical training Interprofessional team rounds Real patients' consultations

*Work based learning appeared to be the least utilized approach… Simulation‐based learning emerged as the most frequent learning approach reported*.

According to the studies reviewed (*N* = 37), medicine and nursing were the most frequent healthcare professions engaged in IPE programmes at 89% (*n* = 33) [12, 17, 18, 19, 20, 21, 22, 23, 24, 25, 26, 28, 29, 30, 32, 33, 34, 35, 36, 37, 38, 39, 40, 41, 42, 43, 44, 45, 46, 47, 49, 50, 51] and 78% (*n* = 29) [12, 17, 18, 19, 20, 21, 22, 23, 24, 25, 26, 27, 28, 29, 31, 32, 33, 34, 35, 36, 38, 39, 40, 41, 44, 45, 46, 48, 50], respectively. Additional healthcare professions that participated in IPE programmes included pharmacy (16%; *n* = 6) [23, 35, 37, 41, 45, 46], respiratory therapy (11%; *n* = 4) [20, 25, 33, 44], psychology (11%; *n* = 4) [37, 45, 46, 48], midwifery (5%; *n* = 2) [24, 34], radiology (5%; *n* = 2) [16, 27], public health (3%; *n* = 1) [30] and medical physics (3%; *n* = 1) [16]. Further, most of the included studies conducted the IPE programme at clinical settings (46%; *n* = 17) [12, 18, 19, 21, 24, 26, 28, 30, 35, 40, 41, 42, 44, 45, 46, 47, 50] and/or at simulation centres (19%; *n* = 7) [16, 25, 29, 32, 34, 36, 39] as depicted in Supporting Information S6.

*Medicine and nursing were the most frequent healthcare professions engaged in IPE programmes*.

To further analyse the synthesized evidence in a manner that addresses both the cognitive and social dimensions of IPE, the model developed by the University of British Colombia (UBC) and the IPE socialization framework were used [9, 10, 11].

**FIGURE 2:**
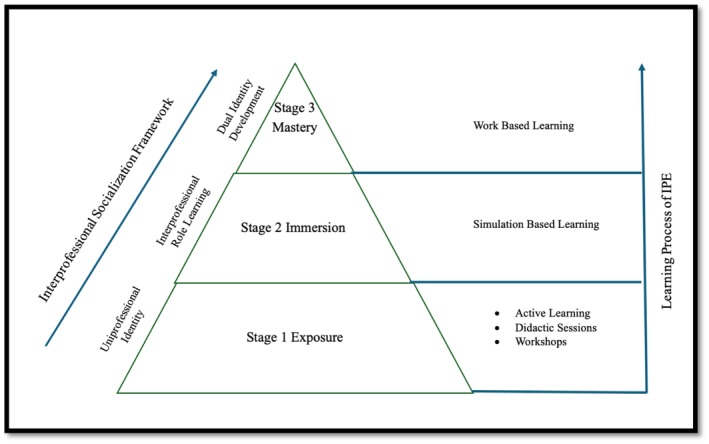
Mapping learning and teaching approaches of interprofessional education (IPE) implemented at postgraduate level for different health professions according to University of British Columbia model and the Khalili's socialization framework of IPE.

Reflecting on the results of this systematic review considering the alignment of UBC Model and IPE Socialization framework reveals that three of the teaching and learning approaches of IPE in postgraduate curricula were at the exposure level, one approach was at the immersion level, and only one learning approach was at the mastery level. Please see Figure 2.

## 4 Discussion

The present systematic review focused on postgraduate studies and explored the available evidence on IPE teaching and learning approaches among healthcare professionals. Based on the evidence, simulation is the most reported IPE teaching and learning approach at the postgraduate level. Moreover, the evidence revealed that active learning was the second most utilized approach, followed by didactic learning, workshops and work‐based learning.

### 4.1 IPE From Undergraduate to Postgraduate

IPE literature highlights the importance of starting interprofessional training from undergraduate to postgraduate levels to prepare health professionals to work collaboratively in diverse and complex healthcare environments. Such training enhances communication, fosters a better understanding of professional roles and improves the efficiency of patient care and the health system. This ongoing education is essential for adapting to the dynamic challenges of healthcare delivery [56]. Certainly, it emphasizes the need for curricula that plan for the transition from one stage of IPE to the other with careful consideration of identity development according to the Interprofessional Socialization Framework [10].

*The need for curricula that plan for the transition from one stage of IPE to the other with careful consideration of identity development*.

### 4.2 Simulation for IPE

The available literature addresses the utilization of many tools to deliver IPE and provides many recommendations to ensure success. For instance, the literature indicates that, at the postgraduate level, simulation is an effective tool for IPE in the education of health professionals. Simulation refers to imitating real‐world processes, systems or situations in a controlled environment for the purpose of training. It offers a range of benefits, including improved patient care, role clarification and the development of communication skills [57]. Furthermore, simulation offers a safe environment for learners to acquire and practice various clinical skills [57]. Simulation is also reported to be an effective method for the development of soft skills such as teamwork, communication, collaboration and leadership [58].

### 4.3 Challenges and Benefits of Using Simulation for IPE

The use of simulation for postgraduate activities presents several challenges, such as scheduling conflicts, logistical issues and high costs. Consequently, the proficient execution of simulations requires a comprehensive evaluation of needs, ensuring that the initiative is suitably incorporated into the curriculum, while also addressing logistical considerations and providing adequate training for the faculty members expected to facilitate the activity [59]. Despite its tactical and strategic considerations and challenges, the benefits of utilizing simulation to deliver IPE sessions to improve patient care are significant. Thus, it is understandable that simulation is the most reported utilized tool in delivering IPE to postgraduate health professionals. The literature also highlights the advantages of role play—a simulation technique that involves using a scenario in which participants act out specific roles—in delivering IPE to health professionals, including enhancing patient outcomes and dismantling traditional barriers to effective teamwork. Furthermore, the literature underscores the importance of role play in IPE in promoting teamwork, collaboration and understanding of professional roles, emphasizing the significance of effective facilitation and the utilization of methods that facilitate the exchange of perspectives during role play [60].

### 4.4 Active Versus Didactic Learning for IPE

Active learning refers to an educational approach where teachers design educational activities to engage students and require them to process and apply information [53]. Studies have shown active learning can significantly enhance students' perceptions of interprofessional teamwork, roles and patient outcomes. For instance, students participating in active learning activities during an IPE week reported significant improvements in these areas compared with those who attended passive, didactic sessions. Active learning methods, such as team‐based learning, are preferred by medical students and are associated with deeper comprehension and retention of material. Additionally, active learning fosters engagement and interaction among learners, enhancing the educational experience [61].

Didactic learning, characterized by focusing on knowledge transmission and content delivery approaches, remains prevalent in many educational settings. While it effectively disseminates basic knowledge to large groups, it often lacks the engagement and interactive elements of active learning. However, didactic methods can still be valuable, particularly when combined with other strategies. For example, a study comparing didactic and flipped classroom methods found no significant differences in student outcomes, although students perceived didactic methods as more beneficial for understanding and retention. Active methodologies and digital tools can enhance didactic sequences to promote meaningful learning [61].

A study has found no significant difference in performance outcomes between active learning and traditional lecture‐based methods, although both strategies improved interprofessional learning outcomes [62]. This finding indicates that while active learning is effective, it may not consistently outperform traditional methods in every context. In other words, both active and didactic learning methods have their roles in IPE at the postgraduate level. Active learning is particularly effective in enhancing engagement and collaborative skills, while didactic learning efficiently conveys foundational knowledge. A blended approach that incorporates elements of both strategies may provide the most comprehensive educational experience, catering to diverse learning preferences and maximizing the benefits of IPE.

### 4.5 Workshops for IPE

Workshops are sessions where individuals or groups gather to engage in hands‐on learning, skill development and collaborative activities. The current literature reports that although workshops are used widely in the education of health professionals, their effectiveness depends on careful planning and consideration of participants' needs. In addition, workshops often require additional planning to ensure that they bridge the gap between theory and practice [63].

### 4.6 Work‐Based Learning for IPE

Work‐based learning constitutes a pedagogical approach that facilitates learners in acquiring practical expertise within authentic environments [55]. Work‐based learning demonstrated effectiveness in delivering IPE; for instance, several studies reported that work‐based learning resulted in a deeper understanding of the roles and skills of different health professionals, promoting better IPC and appropriate referrals in the workplace. This leads to improved patient care and more efficient use of resources. Furthermore, learners participating in work‐based IPE report increased confidence in their clinical skills and ability to work with other health professionals [64]. While evidence supports the effectiveness of work‐based learning, challenges such as profession‐driven education and socialization barriers exist. Addressing these through involving educators from different health specialties and embedding IPE in organizational strategies can facilitate its utilization.

### 4.7 Mapping the Findings With UBC Model and Interprofessional Socialization Framework

Upon aligning the UBC model with the Interprofessional Socialization Framework of IPE [11, 12], the findings of this systematic review reveal that the learning and teaching approaches to IPE within the education of postgraduate health professionals focus predominantly on the initial stage of exposure emphasizing uniprofessional identity. This is noteworthy given that postgraduate learners are assumed to typically enter training with an established professional identity. However, few of the activities are designed or delivered at the immersion and mastery levels, where interprofessional role learning and dual identity development, respectively are the focus (Figure 2).

*IPE within the education of postgraduate health professionals focus predominantly on the initial stage of exposure emphasizing uniprofessional identity*.

In stage one (exposure), learners are prepared for the transformational learning that will take place in stages two and three. This preparation occurs by challenging participants' perceptions of their own profession and the roles played by other healthcare professionals. As this stage requires knowledge acquisition and attitude development, it is understandable that a wide variety of teaching and learning activities that embraces both active and deductive learning were utilized, such as team‐based learning and case‐based learning. In the postgraduate context, this stage should serve re‐examining entrenched professional norms and challenge assumptions about the roles and contributions of other health professionals.

In stage two (immersion), participants collaborate with other professionals and develop their IPE competencies with the aid of teaching and learning approaches such as simulation and role play. In the postgraduate context, this stage represents a transitional phase in which postgraduate learners engage directly with peers from other professions in clinically relevant scenarios. Since the results of this systematic review indicate that simulation was the approach most utilized in delivering IPE to postgraduate health professionals, one can claim that postgraduate IPE focuses on the immersion level. Unfortunately, no evidence suggests that any of presented activities is designed to consciously support sustaining identity transformation.

In stage three (mastery), advanced knowledge and skills should be demonstrated and a sense of belonging to IPE teams established. Thus, competency‐based medical education should be substantially represented. Despite this, few studies delivered IPE utilizing work‐based learning, but none of the reported IPE activities implemented competency‐based learning in designing, delivering or assessing these activities. Considering that this review focused on postgraduate health professionals, where mastery of IPE should be at the advanced level, this finding is concerning because it reveals limitations in preparing postgraduate professionals, particularly those at entry level, to handle the ‘perceptual dip’ effectively. Perceptual dip occurs when novice health professionals initially have a positive perception of IPC and hold idealistic expectations about the functions of the healthcare team. However, their perceptions change as they face real‐world challenges, which affect their professional and interprofessional development [65]. To mitigate this, educators should ensure that postgraduate IPE includes level‐appropriate, theory‐informed interventions that not only develop competencies but also support the evolution of interprofessional identity. By intentionally scaffolding IPE experiences across the exposure, immersion and mastery stages, it is possible to strengthen both the individual and collective readiness of postgraduate professionals to engage in effective IPC.

*Limitations in preparing postgraduate professionals, particularly those at entry level, to handle the ‘perceptual dip’ effectively*.

In summary, simulation‐based education was found to be the most frequently used approach in delivering IPE to different health professionals at the postgraduate level. This finding agrees with the recommendation in the available literature and is, considering the evidence that supports the utilization of this tool for IPE purposes, justified. Moreover, the findings of this review support the findings of the work done by Herath and his colleagues in 2017, which noted that IPE activities were typically based in hospitals. Furthermore, the use of didactic and interactive teaching methods varied significantly when IPE was delivered at the postgraduate level.

Although we systematically reviewed the literature to synthesize information about teaching and learning approaches for postgraduate learners in health professions, several limitations should be acknowledged. First, the variability inherent in the designs, methodologies and outcome measures used across all included studies makes it challenging to synthesize their findings consistently. The variation in postgraduate courses, ranging across several disciplines and specializations, is another factor contributing to possible heterogeneity in the identified teaching and learning methods. Also, the review's overreliance on published literature may induce publication bias, as positive results are likely to be geared towards publishing while neutral or negative outcomes are not. Furthermore, the scope of our review was limited to knowledge, skills and attitudes; thus, other areas integral to a full understanding of the quality of postgraduate education may not have been included. These limitations highlight the need for careful interpretation, and we stress that it is necessary to consider a broader perspective when generalizing conclusions from this systematic review.

## 5 Conclusion

The findings of this review confirm that teaching and learning approaches for delivering IPE at the postgraduate level vary significantly and include both didactive and interactive approaches. That said, simulation‐based learning was reported as the approach most utilized by educators. The evidence synthesized revealed that educators continue to design activities targeting the level of exposure while they should proceed to focus on the levels of immersion and mastery. This insight suggests that the current design and execution of IPE activities require further planning and careful alignment of IPE competencies and their levels.

## Author Contributions

**Nouf Sulaiman Alharbi** **:** conceptualization, methodology, data curation, formal analysis, writing review and editing. **Lujayne Bukhari** **:** methodology, writing – original draft, data curation, formal analysis, writing review and editing. **Noof Khalid Albaz** **:** conceptualization, methodology, writing review and editing. **Abdulrahman Saleh Alraddadi** **:** methodology, conceptualization, data curation, writing review and editing. **Reema Albilehi** **:** methodology, data curation, writing review and editing. **Reem Alkahtani** **:** methodology, data curation, writing review and editing. **Seema Nasser** **:** methodology, data curation, writing review and editing. **Taghreed Alnahedh** **:** methodology, data curation, writing review and editing. **Marwh Gassim Aldriwesh** **:** conceptualization, formal analysis, writing review and editing.

## Ethics Statement

Ethical clearance for conducting this review was attained from King Abdullah International Research Center (KAIMRC), King Saud Bin Abdulaziz University for Health Sciences (RYD‐21‐419812‐122994).

## Consent

The authors have nothing to report.

## Conflicts of Interest

The authors declare no conflicts of interest.

## Supporting information


**Supporting Information S1** Search strategy for identification of articles for the review of postgraduate‐level teaching and learning approaches for interprofessional education in the healthcare professions.

**Supporting Information S2** Data extraction tool designed for Postgraduate IPE Systematic Review

**Supporting Information S3** Original quality checklist for mixed‐methodology case studies and other in‐depth complex designs (Mays N, Robert E, & Popay J, 2001).

**Supporting Information S4** Checklist used for assessing quality of included articles

**Supporting Information S5**Overview of the included studies that implemented interprofessional education (IPE) programs in postgraduate education

**Supporting Information S6** Table 1 Results of the critical appraisal of the articles included in the systematic review

## Data Availability

The datasets used during the current study are available in the Supporting Information of this manuscript.
