# Supplementary material for: Interprofessional Education: A Systematic Review of Educational Methods in Postgraduate Health Professions Programs
Source: Clin Teach. 2025 Jun 19;22(4):e70114. doi: 10.1111/tct.70114 (PMC12179584; doi:10.1111/tct.70114)
Supplement: Supplementary file 1 — Supporting Information S1 Search strategy for identification of articles for the review of postgraduate‐level teaching and learning approaches for interprofessional education in the healthcare professions. [file TCT-22-e70114-s004.docx]

**Additional File 1: Search strategy for identification of articles for the review of postgraduate-level teaching and learning approaches for interprofessional education in the healthcare professions.**

| **Keywords** | **PubMed** | **Science Direct** | **Cochrane Controlled Trial Register** |
| --- | --- | --- | --- |
| “*Interprofessional education*” OR “*multiprofessional education*” AND “*postgraduate*” | 355 | 4,256 | 49 |
| “*Interprofessional education*” OR “*multiprofessional education*” AND “*postlicensure*” | 12 | 4,195 | 45 |
| “*Interprofessional education*” OR “*multiprofessional education*” AND “*postspecialization*” | 0 | 4,211 | 45 |
| “*Interprofessional education*” OR “*multiprofessional education*” AND “*residency*” | 1,224 | 4,226 | 52 |
| “*Interprofessional learning*” OR “*multiprofessional learning*” AND “*postgraduate*” | 188 | 3,014 | 29 |
| “*Interprofessional learning*” OR “*multiprofessional learning*” AND “*postlicensure*” | 8 | 2,985 | 28 |
| “*Interprofessional learning*” OR “*multiprofessional learning*” AND “*postspecialization*” | 0 | 2,990 | 28 |
| “*Interprofessional learning*” OR “*multiprofessional learning*” AND “*residency*” | 511 | 3,005 | 33 |
| **Total for each database** | **2,298** | **28,882** | **309** |
| **Total** | **31,489** | | |
