# Supplementary material for: Interprofessional Education: A Systematic Review of Educational Methods in Postgraduate Health Professions Programs
Source: Clin Teach. 2025 Jun 19;22(4):e70114. doi: 10.1111/tct.70114 (PMC12179584; doi:10.1111/tct.70114)
Supplement: Supplementary file 2 — Supporting Information S2 Data extraction tool designed for Postgraduate IPE Systematic Review [file TCT-22-e70114-s001.docx]

**Additional File 2: Data extraction tool designed for Postgraduate IPE Systematic Review**

|  |  |  | **IPE Program** | | | | | | |
| --- | --- | --- | --- | --- | --- | --- | --- | --- | --- |
|  | **Citation and title of the article** | **Country** | **Participating health professions** | **Settings and contexts** | **Learning and teaching approaches** | **Duration** | **Elective or compulsory?** | **Evaluation method** | **Findings** |
| 1 |  |  |  |  |  |  |  |  |  |
