# Supplementary material for: Interprofessional Education: A Systematic Review of Educational Methods in Postgraduate Health Professions Programs
Source: Clin Teach. 2025 Jun 19;22(4):e70114. doi: 10.1111/tct.70114 (PMC12179584; doi:10.1111/tct.70114)
Supplement: Supplementary file 3 — Supporting Information S3 Original quality checklist for mixed‐methodology case studies and other in‐depth complex designs (Mays N, Robert E, & Popay J, 2001). [file TCT-22-e70114-s006.docx]

**Additional File 3: Original quality checklist for mixed-methodology case studies and other in-depth complex designs (**Mays N, Robert E, & Popay J, 2001).

| 1. Question. Did the paper address a clear research question and if so, what was it? In particular, were complex terms such as ‘hospital at home’, ‘private finance’ defined clearly and unambiguously? |
| --- |
| 1. Design. What was the study design and was this appropriate to the research question? |
| 1. Funding. Who funded the study and what was their perspective? |
| 1. Resource system. In this study, from whom was the innovation said to come? |
| 1. Innovation. What was the nature of the innovation? |
| 1. Context. What was the context of the study? Was this sufficiently well described so that the findings could be related to other settings? |
| 1. User system. Who was receiving the innovation (or to whom was it marketed)? |
| 1. Dissemination mechanism. What (if any) were the elements of the active dissemination process and how did they interact? |
| 1. Implementation mechanism. What (if any) were the elements of the active implementation process and how did they interact? |
| 1. Sampling. Did the researchers include sufficient cases/settings/observations so that conceptual rather than statistical generalisations could be made? |
| 1. Data collection. Was the data collection process systematic, thorough and auditable? |
| 1. Data analysis. Were data analysed systematically and rigorously? Were sufficient data presented? How were disconfirming observations dealt with? |
| 1. Results. What were the main results and in what way are they surprising, interesting, or suspect? Were there any unintended consequences and if so, what were they? |
| 1. Conclusions. Did the authors draw a clear link between data and explanation (theory)? If not, what were the limitations of their theoretical analysis? |
| 1. Reflexivity. Were the authors’ positions and roles clearly explained and the resulting biases considered? |
| 1. Ethics. Are there any ethical reservations about the study? |
