# Supplementary material for: Interprofessional Education: A Systematic Review of Educational Methods in Postgraduate Health Professions Programs
Source: Clin Teach. 2025 Jun 19;22(4):e70114. doi: 10.1111/tct.70114 (PMC12179584; doi:10.1111/tct.70114)
Supplement: Supplementary file 4 — Supporting Information S4 Checklist used for assessing quality of included articles [file TCT-22-e70114-s003.docx]

**Additional File 4: Checklist used for assessing quality of included articles**

The checklist was adapted from **(**Mays N, Robert E, & Popay J, 2001).

| Study title: | | | | |
| --- | --- | --- | --- | --- |
| Criteria | Yes | No | Unclear | Not applicable |
| 1. Question. Did the study/project address a specific question/aim? |  |  |  |  |
| 1. Design. Was the study design explained? |  |  |  |  |
| 1. Funding. Did the paper clarify if the study/project was funded? If yes, by whom? |  |  |  |  |
| 1. Resource system. Was the origin of the study/project stated? |  |  |  |  |
| 1. Innovation. Was the nature/background of the innovation illustrated? |  |  |  |  |
| 1. Context. Was the context of the study/project sufficiently described? |  |  |  |  |
| 1. User system. Were the participants in the study/project elucidated? |  |  |  |  |
| 1. Implementation mechanism. Was the implementation process explained? |  |  |  |  |
| 1. Sampling. Did the authors include sufficient cases/settings/observations so that conceptual rather than statistical generalisations could be made? |  |  |  |  |
| 1. Data collection. Was the data collection process systematic, thorough and auditable? |  |  |  |  |
| 1. Data analysis. Were data analysed systematically and rigorously? Were sufficient data presented? |  |  |  |  |
| 1. Results. Were the main results stated with enough details? |  |  |  |  |
| 1. Conclusions. Did the authors draw a clear link between data and explanation (theory)? |  |  |  |  |
| 1. Reflexivity. Were the authors’ positions and roles clearly explained and the resulting biases considered? |  |  |  |  |
| 1. Ethics. Was the declaration of interest stated? |  |  |  |  |
| Final decision: | | | | |
| Comment: | | | | |
| Reviewer: | | | | |
