# Supplementary material for: Interprofessional Education: A Systematic Review of Educational Methods in Postgraduate Health Professions Programs
Source: Clin Teach. 2025 Jun 19;22(4):e70114. doi: 10.1111/tct.70114 (PMC12179584; doi:10.1111/tct.70114)
Supplement: Supplementary file 5 — Supporting Information S5Overview of the included studies that implemented interprofessional education (IPE) programs in postgraduate education [file TCT-22-e70114-s002.docx]

**Additional File 6: Overview of the included studies that implemented interprofessional education (IPE) programs in postgraduate education**

|  |  |  | **IPE Program** | | | | | | |
| --- | --- | --- | --- | --- | --- | --- | --- | --- | --- |
|  | **Citation and title of the article** | **Country** | **Participating health professions** | **Settings and contexts** | **Learning and teaching approaches** | **Duration** | **Elective or compulsory?** | **Evaluation method** | **Findings** |
| 1 | (Van Schaik et al., 2011)  “Interprofessional team training in paediatric resuscitation: A low-cost, in situ simulation program that enhances self-efficacy among participants” | USA | Paediatric residents: n=82  Paediatric nurses: n=470 | Inpatient and outpatient  settings | Simulation | No mention | Elective | Surveys | Delivering interprofessional team training through utilizing simulation education showed to be feasible and sustainable.  Both participating residents and nurses reported improvement in self-efficacy. |
| 2 | (Salam et al., 2012)  All the World's a Stage: Integrating Theatre and Medicine for Interprofessional Team Building in Physician and Nurse Residency Programs | USA | Nurses and resident  physicians: n= no mention  (45 physician-nurse teams) | Simulation training centre | Simulation | No mention | Elective | Surveys | IPE using simulation helped participants in developing needed clinical and communication skills. |
| 3 | (Lagan et al., 2013)  “Evaluation of an interprofessional clinician-patient communication workshop utilizing standardized patient methodology” | USA | Nurses: n=9  Resident physicians: n=9 | No mention | Clinician–patient communication workshops | 4 hours for  clinical patient communication and 10 minutes for  standardized patient | Elective | Surveys | The importance of communication training and using of standardized patients were valued by the participants in all groups.  In one of the participating groups, the addition of IPE appeared to result in less professional satisfaction. |
| 4 | (Bishop et al., 2015)  “Internists, pharmacists, and psychologists on learning teams: An interprofessional team-based learning experience in graduate medical education” | USA | Internal medicine residents (post graduate year 1 and 3), pharmacy residents (post graduate year 1 and 2), and psychology graduate students: n= no mention | No mention | Team-based learning (TBL) | 3 interprofessional TBL modules were delivered over the course of 1 academic year. | Elective | Pre- and post-surveys | TBL was feasible and effective to deliver IPE.  TBL in IPE can enhance self-perceived knowledge and attitude toward team-  based care. |
| 5 | (Dworetzky et al., 2015)  “Interprofessional simulation to improve safety in the epilepsy monitoring unit” | USA | Neurology residents: n=19  Nurses: n=2 | Centre for medical simulation at the hospital | PowerPoint presentations and scenario-based simulations | One day (3 hours) | Compulsory | Pre-program and post-program knowledge and attitude questionnaires | Significant improvement in knowledge was reported. However, conﬁdence in management was not.  No strong evidence to support that training led to sustainable improvement in performance. |
| 6 | (Thompson Buum et al., 2015)  “Essentials of Ambulatory Care: A postgraduate Level, Interdisciplinary, Interprofessional Curriculum  at the University of Minnesota” | USA | Pharmacy residents: n=24  Internal medicine residents: n=7  Paediatric residents: n=4  Family medicine residents: n=3  Nurse practitioner students: n=8 | Family medicine clinic for the first session.  No mention of other sessions. | Workshops | 1 day workshop, 3 times yearly | No mention | Pre- and post-  workshops  surveys and debriefing | Both faculty and participants perceived the workshop as successful in achieving its objectives.  Logistics of considering IPE such as setting date and finding space were challenging.  The post workshop survey showed a slight drop in interest in primary care (85 to 80%) that could be related to how primary care was presented in the workshops.  Some participants thought the sessions were insignificant because the skills taught were something already delivered to them during medical school. |
| 7 | (Watters et al., 2015)  “Does interprofessional simulation increase self-efficacy: A comparative study” | UK | Nurses, midwives, and medical doctors: n=187 | High-fidelity clinical simulation facility | High-fidelity simulation | 3 days: 2 for debriefing and 1 for the course | Elective | Pre- and post-questionnaires | The post questionnaire showed the following:   - An improved trainees’ confidence and self-efficacy - Nurses and midwives showed more improvement than doctors - Doctors had higher scores in leadership and management - Combined nurse/doctor IPE had positive effects in improving learning outcomes - Doctors showed better final outcomes for communication/teamwork skills |
| 8 | (Chung et al., 2016)  “Interprofessional sepsis education module: A pilot study” | Canada | Emergency medicine residents and family physicians residents: n=15  Emergency registered nurses: n=4  Respiratory therapist: n=2 | No mention | Mixed simulation modality | 2 sessions in February and April 2012 | Elective | The program ended with a  quantitative  evaluation that included the option to provide general free- text feedback.  Online 8  month follow up survey | Participants found the structure of the module was helpful in acquiring knowledge.  A signiﬁcant improvement in pre- and post-  knowledge test scores from 75% to 85%, respectively (p<0.0001) was reported.  The 8-month follow-up survey showed knowledge retention. |
| 9 | (Larson-Williams et al., 2016)  “Interprofessional, multiple step simulation course improves paediatric resident and nursing staff management of paediatric patients with diabetic ketoacidosis” | USA | Paediatric and medicine paediatric residents: n=20  Paediatric nurses: n=25  Graduating residence (as reference group): n=16 | Simulation centre | Scenario based simulation | 17 sessions (once monthly) | Compulsory for endocrinology rotation.  Elective for elective rotations if they are not already  scheduled for endocrinology rotation. | Pre- and post-tests.  Comparison with a reference group not exposed to simulation.  A follow up post-test was completed 3-6 months after the simulation. | Pre-test results were similar in reference group and participants groups.  Participants showed knowledge improvement in the immediate post-test in comparison to the reference group.  In the follow up test, knowledge decay was found.  Participants felt that the IPE and the longitudinal nature of the activity supported their learning. |
| 10 | (Sadideen et al., 2016)  “Using "The Burns Suite" as a novel high fidelity simulation tool for interprofessional and teamwork training” | UK | Clinicians and nurses: n=22 | No mention | High fidelity, team-based simulation | 8 scenarios were run but the duration of conducting the activity and frequency was not mentioned. | Elective | Questionnaire and semi-structured interviews | Participants reported that the simulated scenarios offered an authentic learning experience to improve non-technical skills and IPE.  Participants reported the need for establishing a facility. |
| 11 | (Gupte et al., 2016)  “Together We Learn: Analysing the Interprofessional Internal Medicine Residents’ and Master of Public Health Students’ Quality Improvement Education Experience” | USA | Second postgraduate year at Boston Medical Centre and third postgraduate  year internal medicine residents: n=90  Master of Public Health students enrolled in the lean management and operations management in healthcare courses at Boston University School of Public Health participated in the Quality Improvement curriculum: n=33 | Across schools, departments and the hospital | - Didactic sessions, - Project based-education | 3 weeks inpatient and 1 week ambulatory | No mention | Assessment of attitudes toward IPE through  questionnaire | At the end of the curriculum, residents showed improvement in attitude towards learning and engaging in quality improvement work.  In comparison to residents, Master of Public Health students demonstrated significantly more positive attitudes about IPE. Further, the master students showed stronger agreement than residents that patients would benefit if residents and public health students worked collaboratively. |
| 12 | (Traynor M et al., 2017)  “Why we need more research into interprofessional education” | UK | Doctors: n=6  Nurses: n=6 | Simulation suite | High-fidelity simulation | 1 day | Elective | Pre- and post-questionnaires | IPE session had a positive effect on the participating doctors.  Doctors agreed on the benefits of collaboratively developing team-working skills. |
| 13 | (Keshmiri et al., 2017)  “Effectiveness of an interprofessional education model based on the transtheoretical model of behaviour change to improve interprofessional collaboration” | Iran | Residents of emergency medicine and nurses of the  emergency units: n=91 | Emergency units of two teaching hospitals affiliated to  Iran University of Medical Sciences | - Interactive lectures - Discussion in an IPE small group - Case-based learning - Modified buzz group - Snowball interprofessional discussion - Role-play | No mention | No mention | Assessment of interprofessional collaborative performance by two assessors in each group at 3 and 6 months after the intervention | IPE model that was tailored to the learners' stage of readiness to change showed improvement in interprofessional collaboration among participants. |
| 14 | (Wang et al., 2017)  “Comparative Effectiveness of Hands-on Versus Computer Simulation–Based Training for Contrast Media Reactions and Teamwork Skills” | USA | Radiologists, technologists,  and nurses: n=33  (11 teams of three with one of each) | No mention | High-fidelity  hands on simulation and computer-  based simulation  training | High-fidelity hands-on simulation training: 2 hours  Computer-based simulationtraining: 30-113 minutes | Elective | Experts independently assessing teams’ performance | Training and testing interprofessional  teams of radiologists, technologists, and nurses for both contrast reaction management and teamwork skills is effectively done using high-fidelity hands-on simulation.  High fidelity simulation was preferred over computer-based simulation training by the participants.  Authors found that a single session of either type of training may be inadequate for mastering teamwork skills. |
| 15 | (Blondon et al., 2017)  “Interprofessional collaborative reasoning by residents and nurses in internal medicine: Evidence from a simulation study” | Switzerland | Residents: n=14  Nurses: n=14 | University hospital | Simulation | 28 simulations scenarios with no mention of its period of implementation. | Elective | 3 evaluators, comprising 2 senior physicians and one nurse, evaluated individual and team performance using a five-point Likert scale. | Evidence of collaborative reasoning between residents and nurses, provide explanations to the patient, and team communication, powered by situational awareness.  The results suggested a different reasoning process used by nurses and residents. |
| 16 | (Gilfoyle et al., 2017)  “Improved Clinical Performance and Teamwork of Pediatric Interprofessional Resuscitation Teams with a Simulation-Based Educational Intervention” | Canada | Interprofessional paediatric resuscitation teams including resident physicians, intensive care unit nurse practitioners, registered nurses, and registered respiratory therapists: n=300 (51 teams) | Simulation centres affiliated  with four academic paediatric hospitals | - Simulation based team training - Interactive lectures - Group discussion - Four simulated resuscitation scenarios, each followed by a debriefing | 1 day simulation session | Elective | Pre-post scenarios | A positive correlation between clinical and teamwork performance suggested that effective teamwork improved clinical performance of resuscitation teams. |
| 17 | (Egenberg et al., 2017)  “Impact of multi-professional, scenario-based training on postpartum haemorrhage in Tanzania: A quasi-experimental, pre- vs. post-intervention study” | Tanzania | In 2013:  Nurses and midwives: n=35  Doctors, n=11  Medical attendants: n=24  In 2014:  Nurses and midwives: n=40  Doctors: n=7  Medical attendants: n=20 | Tanzanian Zonal Consultant  Hospital | After technical skill training on the birthing simulator MamaNatal, the teams practiced in realistic scenarios on postpartum haemorrhage.  Each scenario was followed by debriefing and repeated scenario. Afterwards, the group swapped roles and the observers became the participants. | 2-week period in 2013  2-week period in 2014 | No mention | To evaluate the effects of training, the team measured patient outcomes by determining blood transfusion rates. | The results supported that scenario-based training contributed to enhance competence on postpartum haemorrhage management with positive consequences on team efficiency and patient outcome. |
| 18 | (Borman-Shoap et al., 2018)  “Essentials of Ambulatory Care: An Interprofessional Workshop to Promote Core Skills and Values in Team-based Outpatient Care” | USA | Nurse practitioner graduate students: n=99  Internal medicine residents: n=67  Family medicine residents: n=24  Paediatric medicine residents: n=24  Pediatric residents: n=46  Pharmacy residents: n=69 | No mention | - Workshop - Role play - Case-based discussion | No mention | Elective | Pre- and post- workshops surveys | Participants had positive attitude towards a 1-day workshop and active learning.  Participants reported increase confidence in working interprofessionally after the workshops.  Participants showed increase interest in interprofessional collaboration. |
| 19 | (Garber et al., 2018)  “Postpartum Magnesium Sulfate Overdose: A Multidisciplinary and Interprofessional Simulation Scenario” | No mention | Obstetrics and gynaecology and anaesthesiology residents, and labour and delivery nurses: n= no mention | No mention | Simulation | No mention | Elective | Surveys | Participants found that the provided IPE program improved knowledge and practice. |
| 20 | (Truta et al., 2018)  “Improving nontechnical skills of an interprofessional emergency medical team through a one-day crisis resource management training” | Romania | Emergency medicine attending physicians: n=20  Emergency medicine residents: n=10  Nurses: n=40 | Tirgu-Mures  Emergency Clinical County Hospital | - Didactic sessions - Simulation sessions. | 1 day | Elective | Before and after training assessment of teams’ performance through 2 assessors who were blind to the team’s training status | A significant improvement in all professional categories. |
| 21 | (Chang et al., 2019)  “An interprofessional training program for intrahospital transport of critically ill patients: model build-up and assessment” | Taiwan | Residents: n=131  Nurses: n=128  Respiratory therapies: n=16 | No mention | Simulation | 4 months | Elective | Pre- and post- questionnaires and self-reflection | The use of simulation to deliver IPE was found to develop technical and non-technical skills in the participants. |
| 22 | (Rochlen et al., 2019)  “Pilot One-Hour Multidisciplinary Team Training Simulation Intervention in the Operating Room Improves Team Nontechnical Skills” | No mention | Otolaryngology faculty, otolaryngology residents, anaesthesiology faculty, anaesthesiology  residents, nurses, surgical scrub technicians, and perioperative technicians: n= no mention | On site operation room in an adult hospital at a level I trauma centre | Simulation-based team training. | 1 hour  4 intervention sessions were conducted on 4 separate days. | No mention | Trained observers assessed the teams’ nontechnical skills during the intervention and throughout the clinical day.  The participants completed self-reflection surveys before the intervention and in 3 intervals after the intervention. | IPE competencies were not mentioned specifically.  Observers’ assessment showed that team nontechnical skills improved from the first simulation to the second simulation during the intervention and remained higher throughout the clinical day.  Individual self-reflection scores followed the same trend. |
| 23 | (Nicholson et al., 2019)  “Successful implementation of a novel collaborative interprofessional educational curriculum for nurses and residents in a paediatric acute care setting” | USA | Nurses and resident physicians: n=146 | Paediatric acute care unit and urban tertiary care academic children's hospital | Team-building exercise and three high fidelity simulations | No mention | Elective | Pre- and post- surveys | Improved attitude towards IPE.  There were discipline-specific improvements, most notably in the nurses’ response to questions in the domain of teams and teamwork and residents’ response to questions in the domain of interprofessional communication. |
| 24 | (Quatrara et al., 2019)  “Enhancing interprofessional education through patient safety and quality improvement team-training: A pre-post evaluation” | USA | Medical residents: n=32  Doctor of nursing students: n=7 | No mention | Simulation, discussions, Seminars,  Workshops | 7 hours workshops over 3 years | No mention | Pre- and post-Questionnaires | IPE can develop the skills needed to collaborate in providing health care that drive quality improvement and ensures patient safety. |
| 25 | (Ball et al., 2021)  “Radiotherapy-specific interprofessional learning through simulation” | UK | Radiotherapy: n=13  Oncology registrar: n=7  Medical physics trainees: n=6 | Simulation centre | Simulation | No mention | Elective | Readiness for Inter Professional Learning Scale | Simulation in IPE improved communication and team-work skills in participants.  Simulation improved participants interest in IPE. |
| 26 | (Araujo et al., 2021)  “Multiprofessional family health residency as a setting for education and interprofessional practices” | Brazil | Psychology: n=1  Odontology: n=1  Nutrition: n=1  Nursing: n=4  Social Services: n=1  Physical Education: n=1 | Public higher education institution. | - PBL, - Theoretical classes, - In-service practical activities. | March 2017 - February 2019 | Distributed in a module common to all professions. | Qualitative: Electronic form built from the theoretical framework of interprofessional education was used. | Interprofessional education and practice provided opportunities for developing collaborative skills, enhancing teamwork, and interprofessional work. |
| 27 | (Bhattacharya et al., 2021)  “ Preparing a healthcare workforce for geriatrics care: An interprofessional team-based learning program | USA | Dietetics: n=5  Family Medicine -Resident; n=127  Nursing: n=513  Occupational Therapy: n=299  Pharmacy: n=280  Psychology: n=21  Physical Therapy: n=299  Social Work: n=48 | The Geriatrics Champions Program (GCP) | Team-based learning (TBL) | it is a 24-month program, repeated twice.  - five-years program form 2011 to 2016 | Individual programs  determined whether the course was required or elective. | Individual and team Readiness Assessment Tsts (iRAT and tRAT).  Survey were also used to collect feedback.  Wilcoxon signed rank test were used to compare iRAT and tRAT scores. | learners felt this program was helpful for their career preparation in interprofessional geriatric care.  Learners understood that teams performed better than individuals in the care of older adults.  Learners felt better prepared for geriatrics care. |
| 28 | (Liaw et al., 2021)  “ We’re Performing Improvisational Jazz”: Interprofessional Pediatric Palliative Care Fellowship Prepares Trainees for Team-Based Collaborative Practice” | USA | Physician: n=21  Social Work: n=12  Nurse Practitioner: n=8 | Paediatric Advanced Care Team (PACT) Fellowship program. | Clinical training (inpatient and outpatient settings) and daily interprofessional team rounds. | One year  This study included all those who completed the PACT between 2002 and 2018. | No mention. | Survey, qualitative semantic content analysis of fellows' responses, and compared fellow’s ability before and after fellowship. | The educational framework of the fellowship rests on the strengths of  the program’s clinical learning environment and strong interprofessional culture that models collaborative teamwork in a way that helps trainees develop interprofessional competencies. |
| 29 | (Topperzer et al., 2021)  “Postgraduate Interprofessional Case-Based Learning in Childhood Censer: A Feasibility Study” | Denmark | Nurses: n=22  Doctors: n=6  Dieticians: n=2  Pharmacist: n=4  Pharmacologist: n=1  Physiotherapist: n=3  Occupational therapist: n=1  Nurse assistant: n=1  Social Worker: n=1  other specialist:  Daycare worker: 1  Secretary: 2  Teacher: 3  Priest: 2 | Copenhagen University Hospital- Rigs Hospitalet. | Interprofessional Case-based learning sessions | Around 7 weeks | Elective-based on inclusion criteria. | Pre-Post Questionnaires, AITCS, RIPLS, and SAQ.  Also, MCQ.  Finally, at the end case-based Learning session: the participants rated the sessions based on a five-point scale. | The outcome measures suggest that interprofessional case-based learning can potentially influence healthcare professionals’ knowledge of and attitudes towards collaboration and interprofessional learning.  The results of this feasibility study may be useful in planning, designing, and evaluating postgraduate interprofessional education in other settings. |
| 30 | ( Hampton et al., 2022)  “Interprofessional Education Module on Post-Intensive Care Syndrome for Internal Medicine Residents” | USA | Multidisciplinary team (Internal medicine resident physician, a critical care physician, a physical therapist, an occupational therapist, a speech-language pathologist, and a registered dietician. | No mention | Virtual sessions | 3-hour virtual module | Delivered during a mandatory weekly didactic conference for internal medicine residents. | Pre-Post assessment. | Knowledge of PICS and interprofessional roles increased. Integrating interprofessional training in PICS education using virtual platforms may improve residents’ knowledge of interprofessional roles in the ICU and their confidence in managing PICS. |
| 31 | (Meeuwsen et al., 2022)  “ Learning mechanisms and outcomes of an interprofessional molecular pathology workshop for residents) | Netherlands | Clinical scientists in molecular pathology (CSMP) residents: n=10  Pathology residents: n=8  Medical oncology or pulmonary medicine residents: n=4 | No mention | Workshop | No mention | No mention | Pre-post questionnaire and interviews. | All participants learned new technical and medical insights about their and other disciplines.  This workshop in cancer diagnostics was most valuable early during residency since residents came into contact with each other. It increased knowledge and improved IPC between residents in the remainder of their residency. Meetings and collaborative  assignments for medical residents of different disciplines improved  awareness of the importance of IPC. |
| 32 | (Naccarato, et al., 2023)  “A pharmacist-led interprofessional learning experience for family practice medical residents specializing in HIV care.” | Canada | Physicians and Pharmacists. | Ontario HIV Treatment Network partnered with the University Of Toronto Department of Family & Community Medicine. | - Case-based learning, - Real patient consults, and - Experiential and cooperative learning sessions. | One month | Rotation in a six-month residency program. | Kirkpatrick model | The pharmacist-preceptor and their physician colleagues noted improvement in the residents’ pharmacotherapy assessments and therapeutic skills. |
| 33 | (Davis, et al,. 2024)  “Evaluation of Interprofessional Delivery of Diabetes Medication Management Training Among Family Medicine Residents” | USA | The family medicine outpatient clinic is made up of a physician, registered nurse, pharmacist, and pharmacy resident | Midwestern Rural Family Medicine Clinic. | - Lecture-style, - Simulation lab, - Patient case scenarios. | No mention | No mention | Pre-Post surveys. | Of the 13 learners who completed Part 2 of the survey, 100% improved in confidence by at least 1 point.  Implementing small changes to how the education is delivered and how the survey is formatted and administered could effectively enhance learners’ retention and evaluation of knowledge |
| 34 | (Davila et al., 2024)  “Interprofessional Curriculum Delivery: Experience of a Primary Care Education Program” | USA | Health profession trainees, including physician residents, nurse practitioner residents, pharmacy residents, and psychology residents, n=369 | Primary care clinic setting | - Didactics sessions, - Workplace learning, - Reflective practices | No mention | No mention | survey data and the qualitative evaluation of narrative feedback | Qualitative data indicated that profession was associated with differences in perceptions of the curriculum. Findings support the need for the thoughtful consideration of profession-specific identity characteristics when designing interprofessional curricula. |
| 35 | (Stryker et al., 2024)  “The Community Primary Care Champions Fellowship: a mixed methods evaluation of an interprofessional fellowship for physician assistants and physicians” | USA | Fourteen physicians, and a behavioralist with at least two years of post-graduate clinical experience | University of Cincinnati College of Medicine Department of Family and Community Medicine and the Mount St. Joseph (MSJ) University Department of Physician Assistant Studies | - Self-study, - Lectures, - Mentoring, - Community expert discussions | One-year, part-time fellowship | No mention | Pre, post, and one-year follow-up self-assessments of knowledge, attitudes, and confidence  pre- and post- wellness surveys, lecture and discussion evaluations, and midpoint and exit focus groups | Fellows showed significant improvement in 24 of 28 self-assessment items across all content areas post-fellowship, and in 16 of 18 items one-year post-fellowship. They demonstrated reductions in emotional exhaustion and depersonalization post-fellowship and increased confidence in working in interprofessional teams post-fellowship which persisted on one-year follow-up assessments. All fellows completed QI projects and four presented their work at national conferences. Focus group data showed that fellows experienced collaborative, meaningful professional development that was relevant to their clinical work. They appreciated the flexible format and inclusion of interprofessional community experts in evening discussions. |
| 36 | (Marwaha et al., 2024)  “ Improving Healthcare in South Texas Rural Communities: An Interprofessional Initiative for Primary Care Dental and Medical Residents” | USA | Primary care dentists  Family and community medicine residents | The University of Texas Health Science Centre at San Antonio | Virtual workshops,  Case discussion. | Half day. Three 40-minute sessions. | No mention | Pre and post-workshop questionnaires | Workshops enhance learners' knowledge and confidence with an intention to incorporate skills learned during the workshop into practice. |
| 37 | (Sung et al., 2025)  “Improving Critical Care Teamwork: Simulation-Based Interprofessional Training for Enhanced Communication and Safety” | Taiwan | Medical doctors,  Nurses,  Respiratory therapists,  Administrative staff | National Cheng Kung University Hospital | Simulation | No mention | No mention | Mixed-methods approach was used to assess the program’s effectiveness. | Quantitative analysis revealed significant improvement in leadership communication (p = 0.0328) and positive trends in teamwork dimensions such as completion and effective communication. However, global team performance showed only modest numerical gains. (p=0.5201) Qualitative feedback highlighted recurring themes such as unclear task delegation, delayed recognition of patient condition changes, and inconsistent use of communication techniques like call-outs and check-backs |
