# Supplementary material for: Interprofessional Education: A Systematic Review of Educational Methods in Postgraduate Health Professions Programs
Source: Clin Teach. 2025 Jun 19;22(4):e70114. doi: 10.1111/tct.70114 (PMC12179584; doi:10.1111/tct.70114)
Supplement: Supplementary file 6 — Supporting Information S6 Table 1 Results of the critical appraisal of the articles included in the systematic review [file TCT-22-e70114-s005.docx]

**Table 1: Results of the critical appraisal of the articles included in the systematic review**

| Citation | Did the study/project address a specific question/aim? | Was the study design explained? | Did the paper clarify if the study/project was funded? If yes, by whom? | Was the origin of the study/project stated? | Was the nature/background of the innovation illustrated? | Was the context of the study/project sufficiently described? | Were the participants in the study/project elucidated? | Was the implementation process explained? | Did the authors include sufficient cases / settings / observations so that conceptual rather than statistical generalisations could be made? | Was the data collection process systematic, thorough and auditable? | Were data analysed systematically and rigorously? Were sufficient data presented? | Were the main results stated with enough details? | Did the authors draw a clear link between data and explanation (theory)? | Were the authors’ positions and roles clearly explained, and the resulting biases considered? | Was the declaration of interest stated? | Total Score (Out of 15) |
| --- | --- | --- | --- | --- | --- | --- | --- | --- | --- | --- | --- | --- | --- | --- | --- | --- |
| (Thompson Buum et al., 2015) | Yes | NA | Yes | Yes | NA | Yes | Yes | Yes | Unclear | Yes | Yes | Yes | NA | No | Yes | 10 |
| (Watters et al., 2015) | Yes | Yes | Yes | Yes | NA | Yes | Yes | Yes | Yes | Yes | Yes | Yes | Yes | Yes | Yes | 14 |
| (Traynor M et al., 2017) | Yes | Unclear | NA | Unclear | NA | Yes | Yes | Yes | Yes | Yes | Yes | Yes | Yes | No | Yes | 10 |
| (Chung et al., 2016) | Yes | Unclear | No | Yes | Yes | No | No | Yes | Yes | No | No | No | No | No | Yes | 6 |
| (Borman-Shoap et al., 2018) | Yes | NA | Yes | Yes | NA | Yes | Yes | Yes | Unclear | Yes | Yes | Yes | No | No | Yes | 10 |
| (Garber et al., 2018) | Yes | NA | Yes | Yes | NA | Yes | Yes | Yes | No | No | No | No | Yes | No | Yes | 8 |
| (Chang et al., 2019) | Yes | Yes | Yes | Yes | NA | Yes | Yes | Yes | Yes | Yes | Yes | Yes | Yes | No | Yes | 13 |
| (Van Schaik et al., 2011) | Yes | NA | Yes | Yes | NA | Yes | Yes | Yes | Yes | Yes | Yes | Yes | Yes | No | Yes | 12 |
| (Salam et al., 2012) | Yes | NA | No | Yes | NA | Yes | Yes | Yes | No | No | Unclear | No | Yes | No | No | 6 |
| (Lagan et al., 2013) | Yes | Yes | No | Yes | NA | No | Yes | Yes | Yes | Yes | Yes | Yes | Yes | No | No | 10 |
| (Dworetzky et al., 2015) | Yes | Yes | Yes | Yes | NA | Yes | Yes | Yes | Yes | Yes | Yes | Yes | Yes | Yes | Yes | 14 |
| (Larson-Williams et al., 2016) | Yes | Yes | No | Yes | NA | Yes | Yes | Yes | Yes | Yes | Yes | Yes | No | No | No | 10 |
| (Sadideen et al., 2016) | Yes | Yes | No | Yes | NA | Yes | Yes | Yes | Yes | Yes | Yes | Yes | No | No | No | 10 |
| (Gupte et al., 2016) | Yes | Unclear | Yes | Yes | NA | Yes | Yes | Yes | Yes | Yes | Yes | Yes | Yes | No | Yes | 12 |
| (Keshmiri et al., 2017) | Yes | Yes | Yes | Yes | NA | Yes | Yes | Yes | Yes | Yes | Yes | Yes | Yes | No | Yes | 13 |
| (Wang et al., 2017) | Yes | Unclear | Yes | Yes | NA | Yes | Yes | Yes | Yes | No | Yes | Yes | No | No | Yes | 10 |
| (Blondon et al., 2017) | Yes | Yes | Yes | Yes | NA | Yes | Yes | Yes | Yes | Yes | Yes | Yes | Yes | No | No | 12 |
| (Gilfoyle et al., 2017) | Yes | Yes | No | Yes | NA | Yes | Yes | Yes | Yes | Yes | Yes | Yes | Yes | No | No | 11 |
| (Egenberg et al., 2017) | Yes | Yes | No | Yes | NA | Yes | Yes | Yes | Unclear | Yes | Yes | Yes | Yes | No1 | Yes | 9 |
| (Truta et al., 2018) | Yes | Yes | No | Yes | NA | Yes | Unclear | Yes | Unclear | Yes | Yes | Yes | Yes | Yes | No | 10 |
| (Rochlen et al., 2019) | Yes | Unclear | No | Yes | NA | No | Yes | Yes | Unclear | Yes | Yes | Yes | No | No | No | 7 |
| (Nicholson et al., 2019) | Yes | No | Yes | Yes | NA | Yes | Yes | Yes | Yes | Yes | Unclear | Yes | No | No | Yes | 10 |
| (Ball et al., 2021) | Yes | No | No | Yes | NA | Yes | Yes | Yes | Yes | Yes | Yes | Yes | Yes | Yes | Yes | 12 |
| (Bishop et al., 2015) | Yes | NA | No | Yes | NA | Yes | Yes | Yes | No | No | No | No | No | No | No | 5 |
| (Quatrara et al., 2019) | No | No | No | Yes | NA | Yes | Yes | Yes | No | Yes | Yes | Yes | Yes | No | Yes | 9 |
| (Araújo et al., 2021) | Yes | Yes | No | Yes | NA | Yes | Yes | Yes | Yes | Yes | Yes | Yes | Yes | No | Yes | 12 |
| (Bhattacharya et al., 2021) | Yes | No | Yes | Yes | NA | Yes | Yes | Yes | Yes | Yes | Yes | Yes | Yes | No | Yes | 12 |
| (Liaw et al., 2021) | Yes | Yes | Yes | Yes | NA | Yes | Yes | Yes | Yes | Yes | Yes | Yes | Yes | No | Yes | 13 |
| (Topperzer et al., 2021) | Yes | No | Yes | Yes | NA | Yes | Yes | Yes | Yes | Yes | Yes | Yes | Yes | No | Yes | 12 |
| (Hampton et al., 2022) | No | No | No | Yes | NA | No | Yes | Yes | No | Yes | Yes | Yes | Yes | No | Yes | 8 |
| (Meeuwsen et al., 2022) | Yes | Yes | No | Yes | NA | Yes | Yes | Yes | Yes | Yes | Yes | Yes | Yes | No | Yes | 12 |
| (Naccarato et al., 2023) | Unclear | No | Yes | Yes | NA | No | Yes | Unclear | No | Yes | No | No | No | No | Yes | 5 |
| (Davis et al., 2024) | Yes | No | Yes | Yes | NA | No | Yes | Yes | No | Yes | Yes | Yes | Yes | No | Yes | 10 |
| (Davila et al., 2024) | Yes | Yes | Yes | Yes | NA | Yes | Yes | Yes | Yes | Yes | Yes | Yes | Yes | No | Yes | 13 |
| (Stryker, et al., 2024) | No | Yes | Yes | Yes | NA | Yes | Yes | Yes | Yes | Yes | Yes | Yes | Yes | No | Yes | 12 |
| (Marwaha et al., 2025) | Yes | Yes | Yes | Yes | NA | Yes | Yes | Yes | Yes | Yes | Yes | Yes | Yes | No | No | 12 |
| (Sung & Hsu, 2025) | Yes | Yes | No | Yes | NA | Yes | Yes | Yes | Yes | Yes | Yes | Yes | Yes | Yes | Yes | 13 |

^*^ Not applicable
